# Nutritional and antinutritional composition of the five species of aquatic edible insects consumed in Manipur, India

**DOI:** 10.1093/jis/14.1.14

**Published:** 2014-01-01

**Authors:** T. Shantibala, R. K. Lokeshwari, H. Debaraj

**Affiliations:** Institute of Bioresources and Sustainable Development, Department of Biotechnology, Government of India, Takyelpat-795001, Manipur, India

**Keywords:** minerals, proximate composition

## Abstract

The people living in Manipur have a distinct identity, culture, and food habits. They have a prototype culture of eating insects. In our study, the nutritive contents of five potentially-edible aquatic insects, *Lethocerus indicus* (Lepeletier and Serville) (Hemiptera: Belostomatidae), *Laccotrephes maculatus* (F.) (Nepidae), *Hydrophilus olivaceous* (F.) (Coleoptera: Dytiscidae), *Cybister tripunctatus* (Olivier), and *Crocothemis servilia* (Drury) (Odonata: Libellulidae), were analyzed to inform consumers about the nutritional quality of the insects and the suggested quantity of their intake. A good amount of protein content and high gross energy was recorded among the insects. The results showed high levels of sodium, calcium, and magnesium present in the insects, indicating that they are a good source of minerals. Antinutritional properties of these insects were below 0.52%, which is a non-toxic level. Aquatic insects, such as *C. tripunctatus* , also possesses strong antioxidant activity (110 µg/mL). Therefore, these insects can play a major role in food security, health, and environment management. It is essential to cultivate edible insects to maintain their population sustainability.

## Introduction

Many species of insects serve as traditional foods among indigenous peoples and they play an important role in human nutrition. Attempts have been made in the past to ensure adequate nutritional and functional quality food supplements are affordable to a target population using the staple commodities of a region ( Orr 1997 ). Traditionally-consumed unconventional food items may supplement the dietary requirement of a population, thus preventing the development of a wide range of diseases associated with malnutrition and others ( Mishra et al. 2003 ). Generally, edible insects represent significant biological resources that are rich in protein, amino acids, fat, carbohydrates, various vitamins, and trace elements ( Xiaoming et al. 2010 ). It has been hypothesized that a 10% increase in the world supply of animal protein through mass production of insects would largely eliminate the malnutrition problem and also decrease the pressure on other protein sources ( Robert 1989 ). Therefore, insects offer an important nutritional resource for humans and are worthy of development in various bio-prospecting aspects ( Xiaoming and Ying 1999 ).

People of many ethnic origins living in Manipur capture and consume many insect species located in puddles, ponds, lakes, rivers, etc. Among the edible insects, aquatic insects are one of the most favorable groups among consumers due to their taste and high availability. During the rainy season, there is an abundance of aquatic insects in various inland water bodies. In the valley region of the state, there are many inland freshwater lakes that act as a source of aquatic edible insects.

While eating insects is common practice in the region, there is little information about the nutritive value of these edible insects. The nutritive values of these insects will inform consumers about the quality and ideal quantity of insect intake. In the meantime, this information can be utilized in the development of nutritional food supplements. Our study was designed to evaluate the nutritive content of five potential aquatic edible insects, *Lethocerus indicus* (Lepeletier and Serville) (Hemiptera: Belostomatidae), *Laccotrephes maculatus* (F.) (Nepidae), *Hydrophilus olivaceous* (F.) (Coleoptera: Hydrophilidae), *Cybister tripunctatus* (Olivier), and *Crocothemis servilia* (Drury) (Odonata: Labullidae). The taxonomic position, consumption stage, and method of preparation are noted ( Table. 1 ).

**Table 1.**
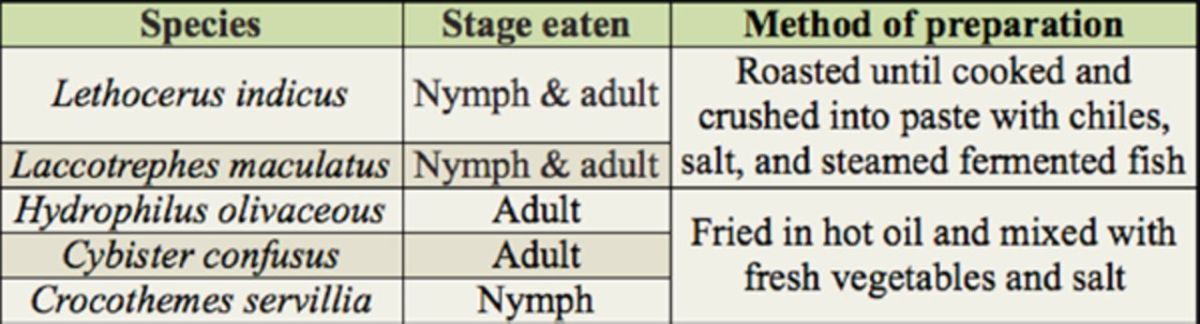
Stage eaten and methods of preparation of edible aquatic insects.

## Materials and Methods

### Assays

The insects were collected from the ponds and submerged paddy fields and adjoining areas of Tangkham Lamphel Pat, Khundrakpam, Imphal East District, Manipur, India, situated 16 km from the Institute of Bioresources and Sustainable Development Imphal.

Nitrogen was determined by the micro-Kjeldahl method ( Pearson 1976 ). To obtain true protein content, the non-protein nitrogen was extracted with icecold 10% TCA and titration against the standard acid. The calculated non-protein nitrogen value was deducted from the total nitrogen content and then multiplied by the factor, 6.25, to determine the actual crude protein content.

The moisture content of the sample was estimated following the method of Hart and Fisher (1971) . Freshly-collected aquatic insects were weighed and kept in an incubator at 66° C until a constant weight was obtained. The difference between initial and final weight was calculated, and the moisture content was represented in percentage.

The carbohydrate content was determined by the Anthrone method ( Dubois et al. 1956 ). Total crude fat content was determined by homogenizing and soaking the sample with chloroform-methanol mixture (2:1 v/v) for around 3-4 hours. Then 0.6% NaCl was mixed at the ratio of 1:1 to the filtrate and left overnight to obtain a clear solution. The sample was then dried, giving the lipid content of the sample ( Folch et al.1956 ). The ash content was estimated by using a muffle furnace (NSW Model-103, www.nsw-inda.com ) at 600° C to constant weight. The crude fiber content was estimated following the method of AOAC (1990) . Two grams of fat-free, powdery form of each sample was heated with 200 mL of 0.25 N sulphuric acid solution for 30 minute and filtered using a Buchner funnel. Then, after washing with distilled water, the residue was again boiled with 200 mL of 0.313 N sodium hydroxide for 30 min. The filtered residue was washed with boiling water. It was washed again with 10% HCl and twice with ethanol. The residue was dried in an oven overnight at 100° C. After cooling, it was ignited in a muffle furnace at 500° C for three hours to obtain the weight of the ash ( AOAC 1990 ). The energy content was estimated using a digital bomb calorimeter, model RSB-3/5/6/6A (Rajdhani Scientific Instrument) designed in accordance with the specifications of the Institute of Petroleum and British Standard Institution (IS: 1350-1966).

For the energy estimation, the known weight of the pellet sample was placed inside the “bomb,” which was then sealed with oxygen. Then the sample was ignited electrically. The heat released during complete oxidation of the compound was measured through the temperature change in the water bath surrounding the bomb by a digital sensor. The heat of combustion at a constant volume was calculated from the resulting rise in temperature using the formula:

$\text{CVs} = \text{T}x\text{W} - (\text{CVt} - \text{CVw})/\text{M}$

where, T = final rise in temperature in °C, M = mass of sample in grams, W = water equivalent in calories per ° C, CVt = caloric value of thread (2.1/cm), CVw = caloric value of ignition wire (2.33/cm), and CVs = caloric value of sample.

The mineral content was determined after wet digestion of sample with a mixture of sulfuric, nitric, and perchloric acids at the ratio of 1:10:4 using an atomic absorption spectrophotometer (model AAS-200/Analyst Version 6, PerkinElmer, www.perkinelmer.com ).

Tannin content was determined by the qualitative method using tannic acid as standard solution ( Enujiugha and Ayodele-Oni 2003 ). A finely-ground sample (0.2 g) was soaked in 10 mL of 70% acetone for 15 minutes in ice water. To the filtrate, 0.5 mL of lowery reagent and 2.5 mL of 20% sodium carbonate was added and incubated for 40 minutes. Absorbance was measured at 700 nm Total phenolic content was estimated by using Fo-lin-Ciocalteu reagent ( Kahkonen 1999 ).

The antioxidant potential of the methanol extract was determined on the basis of their scavenging activity of s Table 1 ,1-diphenyl-2-picryl hydrazyl (DPPH) free radicals ( Sánchez-Moreno et al. 1998 ). Ascorbic acid was used as the standard, and the absorbance was measured at 517 nm. The IC _50_ value denotes the concentration of the sample required to scavenge 50% of the DPPH free radicals.

### Statistical analysis

The data obtained for proximate composition, mineral, and antinutritional values were analyzed with oneway ANOVA. Comparison between mean was made by Tukey’s HSD test. Data are reported as the mean ± SEM. A significance level of 0.05 was used to reject the null hypothesis. Data analysis was done with the help of Statistica Version10 (StatSoft, www.statsoft.com ).

## Results

### Proximate compositions

Parameters such as moisture, crude protein, carbohydrate, lipid, ash, fiber, and energy were analyzed for five species of aquatic edible insects, and the results are presented in Table 2 . There were significant differences in the means of the proximate compositions among the insect species *(p* < 0.05). Regarding moisture content, *H. olivaceous* showed the highest percentage of moisture while *C. servilia* had the least value. There was a significant variation in mean value of moisture content among the five different insects at **a** = 0.05. The level of protein content of *C. servilia* was found to be higher than the other species. Overall, a good amount of protein content was noticed among the aquatic edible insects. A significant variation of protein content was observed at 5% level of ANOVA. However, there was insignificant variation in the percentage of carbohydrates among the aquatic insects. The percentage was lowest in *L. maculatus* and highest in *H. olivaceous.* It was observed that all the five insect species contained a low amount of carbohydrates. There was no significant difference in total lipid content among the *H. olivaceous, L. maculatus,* and *C. servilia* ( **a** = 0.05), but there was a significant difference between *L. indicus* and *C. tripunctatus.* The highest percentage of lipid content was observed in *C. tripunctatus* compared to the other four species. The energy available in the carbohydrates, protein, and fat was also analyzed. A total of about 563.84 kcal/100 g of energy was provided on average per insect. The highest amount of energy was found in *L. indicus,* while the lowest was found in *C. servilia.* A significant variation of fiber content was observed among the aquatic edible insects, except for *C. tripunctatus* and *H. olivaceous.* The highest ash content, which was observed in *C. tripunctatus,* showed insignificant variation with the amount of ash content in *L. indicus.* The other three aquatic insects *H. olivaceous, L. maculatus* and *C. servilia* showed non-significant ash content.

**Table 2.**
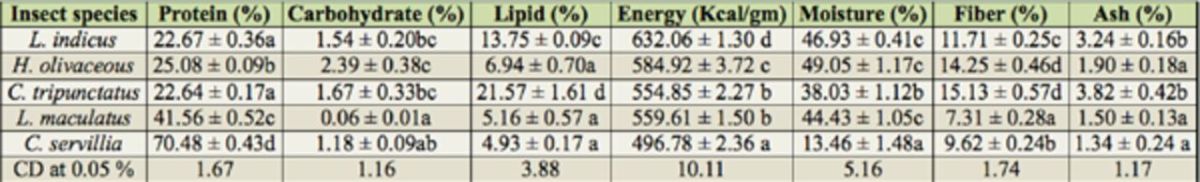
Proximate composition of aquatic edible insects.

All values are mean ±SE. Means followed by the same alphabet in the same column are not significantly different *(p* < 0.05).

### Mineral profile

Out of the micro-nutrient compositions, sodium was the most prominent in *L. indicus, H. olivaceous, L. maculatus* and *C. servilia* ( Table 3 ). There was a significant difference among the species *(p* 0.05). Potassium was the most prominent nutrient observed in *C. tripunctatus.* In general, a high amount of calcium and magnesium were noticed in all the insects. The calcium content was lowest in *H. olivaceous* and highest in *L. indicus,* whereas magnesium lowest in *C. tripunctatus* and highest *H. olivaceous.* No significant difference of iron concentration was observed between *C. servilia* and *C. tripunctatus,* but there were significant differences among the remaining species at a 5% probability level. The zinc content was lowest in *C. tripunctatus* and highest in *L. indicus.* The copper content was relatively low in all species except *L. maculatus.*

**Table 3.**
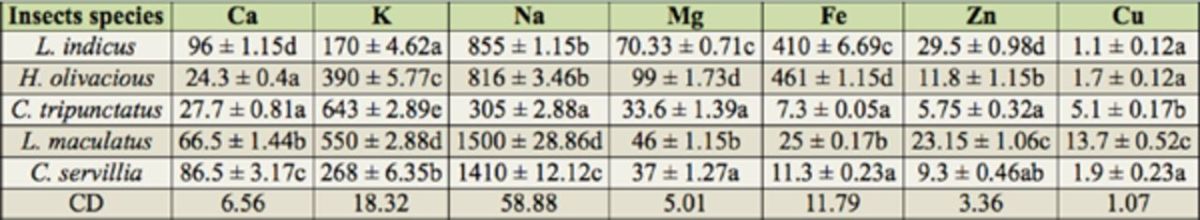
Major mineral profile of different aquatic insects (concentration in mg/100 g).

All values are mean ±SE. Means followed by the same alphabet in the same column are not significantly different *(p* 0.05).

### Antinutritional factor

The estimations of antinutritional factors such as tannin and phenol are presented in Table 4 . The phenol and tannin concentrations of the species were not significantly different from one another. The value of antinutritional parameters was below 0.52% in all species.

**Table 4.**
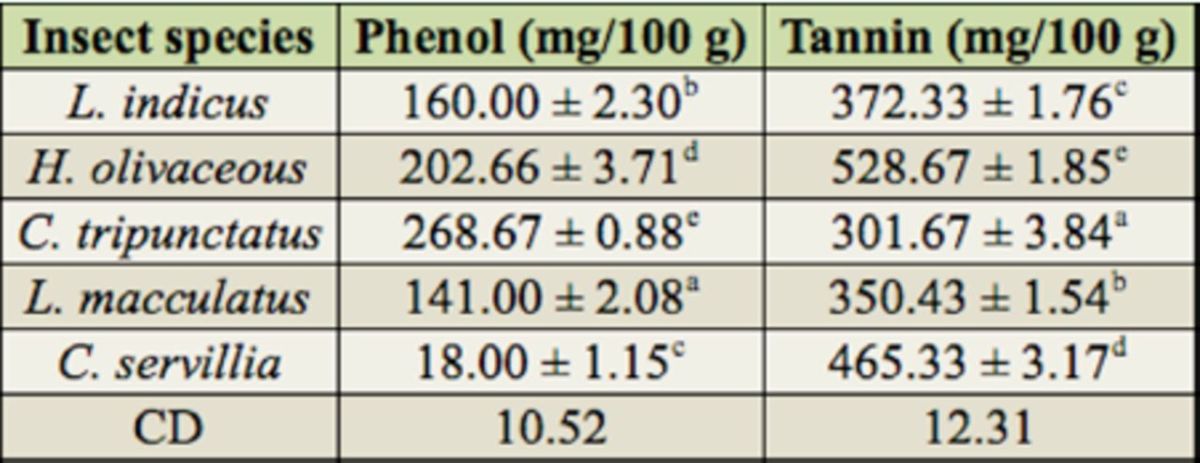
Antinutritional factors of edible aquatic insects.

All values are mean ±SE. Means followed by the same alphabet in the same column are not significantly different *(p* 0.05).

### Antioxidant properties

**Figure 1.**
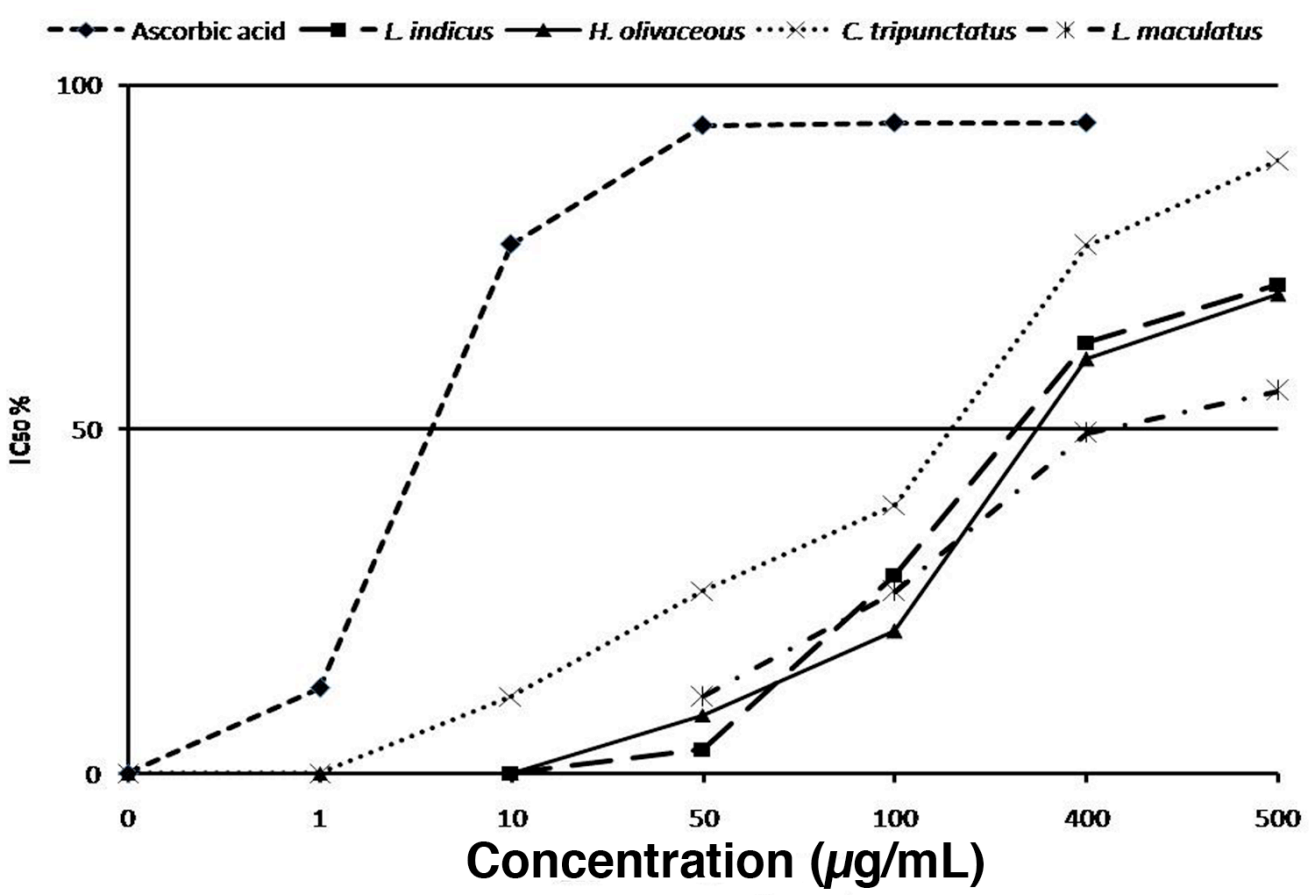
DPPH scavenging assay of aquatic edible insects compared with standard ascorbic acid (IC50% vs. concentration). High quality figures are available online.

DPPH free radical scavenging assay of methanol extract of the aquatic edible insects compared with standard ascorbic acid was analyzed and is presented in Figure 1 . The IC _50_ % of the insects ranged from 110 ( *C. tripunctatus* ) to 880 µg/mL ( *C. servilia* ). The species with lesser IC _50_ % values had stronger antioxidant properties _._ Therefore, among the species, *C. tripunctatus* had the best antioxidant property. The IC _50_ % of *C. servilia* was 880 µg/mL, which was comparatively higher than the other four species but is not a strong antioxidant property.

## Discussion

The present study examined insects typically consumed by humans in Manipur, India. The practice of entomophagy had also been reported in all seven states of the Northeast region ( Pathak and Rao 2000 ; Chakravorty 2011 ) and in other countries such as Thailand ( Hanboonsong 2008 ) and Mexico ( Ramos-Elorduy et al. 2009 ). Consumption of these aquatic insects is common because people tend to use insects that are readily available, plentiful, and easy to capture, store, and prepare for eating.

The insects studied showed high amounts of nutritional content *.* The protein content, which was obtained after removal of the non-protein nitrogen, was comparable with that found in *Ephemeroptera* (66.26 %), *Odonata* (40– 65%), *Hemiptera* (42–73%), and *Coleoptera* (23–66%) by Xiaoming et al. (2008). The protein content (%) exhibited by the insects was significantly higher than in conventional animal meats, and therefore insects may offer an affordable source of protein to counteract protein malnutrition (Kariuki 1991). The carbohydrate content of these aquatic insects (< 2.39%) was within the range of 1–10% reported in edible insects (Xiaoming et al. 2008). The fat content of edible insects lies between 10 and 50% ( Ying et al. 2001 ; DeFoliart 1991). The high fat content of the diving beetle, *C. tripunctatus* (21.57%) can contribute significantly as a source of oil in diets. The fatty acid of edible insects is different from other animal fats, as it has higher fatty acids that the human body needs (Xiaoming et al. 2008). Detailed studies on the oils in edible insects, such as sterol content and saturated and non-saturated fatty acids, are a topic for future research. The crude fiber contents of the insects in this study were quite high, similar to other edible insects such as termites (29.58 g/kg) ( Adeduntan 2005 ) and *Camponotus* sp. (15.95%) ( Mbah and Elekima 2007 ). High crude fiber content in the insects could be due to the chitin normally found in insects ( Akinnawo and Ketiku 2000 ). The high crude fiber content can be used to complement animal roughages in addition to other uses mentioned earlier ( Mbah and Elekima 2007 ). The gross energy value given by these edible insects depends on the amount of protein, fat, and carbohydrate contents in the insect. All the studied species had high gross energy values, a finding similar to the high caloric content value reported from 78 other species of edible insects ( Ramos-Elorduy et al. 1997 ). Because the ash content of a sample is a reflection of the minerals it contains ( Adeyeye 2000 ), these insects were therefore found to be rich in minerals.

The level of minerals present in edible insects indicates that insects are good sources of minerals for the human body ( Kinyuru et al. 2010 ). Sodium was the highest among the macro minerals. Most insects with detritivorous, predaceous, and blood-sucking feeding habits have higher concentrations of sodium than phytophagous insects ( Chapman 1998 ). Because sodium is both an electrolyte and mineral, it helps to maintain the amount of fluid inside and outside the body’s cells and the electrolyte balance in the body. The potassium content of these aquatic insects was very high, so they could be a good source of potassium in diets. The amount of magnesium in these insects was higher than in conventional food items such as bajra (0.137%) and soybeans (0.175%). Magnesium is essential in maintaining both the acid-alkali balance in the body and the healthy functioning of nerves and muscles ( Paul and Dey 2011 ). *Lethocerus indicus* had considerably higher amounts of iron as compared to other insects, such as *Bombyx mori* (1.8 mg/100 g) ( Dunkei 1996 ) and *Cirina for da* (5.34 mg/100 g) ( Omotoso 2006 ). The insects were also a better source of iron compared to the main available sources, such as red meats ( Williams 2007 ). The concentration of zinc in these insects was comparable to those in terrestrial insects ( Kinyuru et al. 2010 ). The amount of calcium in aquatic insects (24.3-96 mg/100 g) is much higher than in different terrestrial insects (0.0012-0.126 mg/100 g) ( Adeduntan 2005 ). The high amount of calcium content in insects could be used as a supplement for children and adolescents who are still developing their bones and teeth. Minerals such as selenium and manganese were reported to present in trace amounts in some other edible insects such as *Cirina forda* ( Omotoso, 2006 ) and bees ( Finke, 2005 ). The antinutritional factors of these insects were found to be low. The tannin in other edible insects, such as locust, ants, termites, and grasshoppers, were higher ( Adeduntan 2005 ; Hassan et al. 2008 ) than the insects in our study. Tannins have traditionally been considered antinutritional, but it is now known that their beneficial or antinutritional properties depend upon their chemical structure and dosage ( Muller-Harvey and McAllan 1992 ). Oral ingestion of 6% diet of the tannin punicalagins for 37 days was found to show non-toxic effects in rats ( Cerdá et al. 2003 ). Phenol causes many subtle effects to the biota, such as reduced fertility, decreased survival of the young, and inhibition of growth ( Babich and Davis 1981 ). A minimal lethal oral dose of phenol has been estimated for adults at approximately 70 mg/kg ( ATSDR 2011 ). It can metabolize readily through the process of eating. The phenol content in the aquatic insects was below the lethal dose. Consumption of these insects will not cause any harmful effect.

In addition to the macro- and micro-nutrient contents, antioxidant properties were also observed in these insects. Among them, *C. tripunctatus* possessed strong antioxidant activity, but it was lower than the antioxidant activity of the plant extract of *Calotropis procera* (121.25 ug/mL) ( Yesmin et al. 2008 ). Edible insects can be considered a quality food item that can enhance the maintenance of health and protect from aging related diseases ( Hsu 2006 ). However, despite all the environmental and nutritional advantages entomophagy offers, it is unlikely to become a mainstream dining option in the near future. The key factor will be in understanding and raising awareness of the potential contributions that edible insects can make to the environment, nutrition, and people’s livelihoods. In many parts of the world where eating insects has been a common element of traditional culture, the practice is waning due to modernization and changing attitudes. In these areas, reviving the tradition of eating insects has significant potential to improve rural livelihoods, enhance nutrition, and contribute to sustainable management of insect habitats ( Durst and Shono 2008 ).

### Conclusion

The existence of the culture of eating insects in Manipur ensures nutritional needs of the indigenous people are being met. Although the use of edible insects has been trivialized, they can play a major role in food security, health, and environment management. Edible insects are rich in protein, fat, carbohydrates, minerals, and other activated elements that promote human health. Insects are characterized by rich species diversity and large populations, therefore as nutritive resources, they can be widely exploited and have great development potential. It is also necessary to cultivate important edible insect species to sustain them.
